# Editorial to “Efficacy of an alternative positioning of intracardiac defibrillation catheters in atrial fibrillation ablation”

**DOI:** 10.1002/joa3.70066

**Published:** 2025-04-15

**Authors:** Hideharu Okamatsu, Ken Okumura

**Affiliations:** ^1^ Division of Cardiology Saiseikai Kumamoto Hospital Kumamoto Japan; ^2^ Department of Cardiology and Nephrology Hirosaki University Graduate School of Medicine Hirosaki Japan

**Keywords:** atrial fibrillation, catheter ablation, intracardiac defibrillation

Atrial fibrillation (AF) is the most prevalent atrial arrhythmia in developed countries. With the increase in the aging population, the number of patients with AF has been increasing. Catheter ablation has become a widely used treatment for AF, with pulmonary vein (PV) isolation (PVI) being the standard approach. As the technologies of ablation advance, complete PVI can be achieved within a shorter procedure time. In radiofrequency catheter ablation, lesion size markers incorporating contact force, radiofrequency application power, and radiofrequency application time enable the operator to create PVI lesions efficiently. Cryoballoon ablation is another technology that allows PVI more easily. Pulsed‐field ablation is a new technology that accomplishes PVI without causing collateral organ damage and PV stenosis. With the progress of these ablation technologies, many operators have streamlined the procedure workflow to reduce procedure time and minimize procedure‐related complications. Internal jugular vein puncture has been performed to advance the electrode catheter into the coronary sinus (CS) to evaluate the anatomy of the CS, record the left atrial and CS potentials, and perform cardioversion to convert AF to sinus rhythm with the use of specific intracardiac defibrillation catheters (ICDC). However, with the advancement of ablation technologies, some operators insert the electrode catheter into the CS via the femoral vein and inferior vena cava (IVC) instead of the internal jugular vein and superior vena cava (SVC) to avoid internal jugular vein puncture, which has some risk of complications, including vascular injury, hematoma, and pneumothorax, and simplify the workflow. BeeAT via IVC approach (Japan Lifeline, Tokyo) is an ICDC designed to insert the electrode catheter into the CS via the IVC. In performing cardioversion, the operator is recommended to insert the distal part of electrodes in the CS and locate the proximal part in the right atrium (RA), forming an alpha‐loop configuration (CS/RA configuration). The operator sometimes needs to insert the distal part into the CS deeply to position the proximal part in the RA. However, inserting the distal part deeply into the CS to make an alpha‐loop configuration is difficult in some patients because of variations in the location and configuration of the CS ostium. Moreover, unintentional insertion of the distal part of electrodes into the branch of the CS may result in venous perforation and cardiac tamponade. Thus, placement of the ICDC in the CS and RA is sometimes challenging and time‐consuming, needs extra fluoroscopy, and causes a risk of CS perforation.

Ohashi et al. studied the efficacy of the new ICDC configuration in performing cardioversion by evaluating 81 patients undergoing cardioversion with ICDC during the AF ablation procedure.^1^ They initially evaluated the ICDC configuration, inserting the distal part of electrodes in CS and locating the proximal part in IVC (CS‐only configuration). However, cardioversion using this CS‐only configuration successfully converted AF into sinus rhythm only in 9 of the 81 patients (11%). Subsequently, they evaluated the following ICDC configuration, locating the distal part of electrodes in the SVC and the proximal part in the IVC (SVC configuration). The cardioversion using this SVC configuration successfully converted AF into sinus rhythm in 67 of the remaining 72 patients (93.1%). This SVC configuration was easily accomplished with minimum fluoroscopy and minimum risk of cardiac perforation. Compared to the RA/CS configuration, this new SVC configuration seems to simplify the procedure workflow and reduce procedure‐related complications.

The SVC configuration has an important limitation. In this configuration, almost all of the proximal parts of electrodes are positioned in the IVC instead of the RA. Consequently, the SVC configuration cannot allow the recording of any triggers from non‐PV origins. Pinpointing the non‐PV origins by recording the premature atrial contraction leading to AF just after defibrillation is essential to ablate the non‐PV origins and to improve the outcome of the ablation procedure, especially in persistent AF. From this standpoint, the SVC configuration offers no clinical advantage over the patch‐based external cardioversion. The patch‐based external cardioversion effectively performs cardioversion without fluoroscopy. Naturally, cardioversion with patch‐based external cardioversion carries a risk of skin burns. However, many patients undergo cardioversion only a few times and have rarely experienced skin burns. A few patients have to undergo cardioversion repeatedly to determine the origin of the non‐PV AF triggers; however, in these patients, neither the ICDC with SVC configuration nor the patch‐based external cardioversion is of value in evaluating the non‐PV triggers, and the ICDC with RA/CS configuration is necessary to determine their locations.

When performing the AF ablation procedure under electroanatomical mapping (EAM), the ICDC offers a clinical advantage over external cardioversion. The ICDC achieves cardioversion using lower energy than external cardioversion, which minimizes patient movement during the procedure and a 3D map shift in the EAM system. For instance, when analyzing the location of the residual conduction gap after linear radiofrequency application around the PVs, a 3D map shift after cardioversion may distort the spatial relationship between the radiofrequency application point tagged in the EAM and the residual conduction gap shown by the EAM and may complicate the procedure in localizing the residual conduction gap. Using the ICDC with SVC configuration allows cardioversion to be performed easily and safely with minimum patient movement during the procedure. Of course, inserting the ICDC into CS via SVC can also allow intracardial cardioversion using lower energy, although an approach from the internal jugular vein is required. Even with these limitations, the SVC configuration may be a useful alternative in simplifying the AF ablation procedure workflow.

## CONFLICT OF INTEREST STATEMENT

Ken Okumura received honoraria from Johnson and Johnson and Medtronic.

## References

[1] Ohashi J , Hayashi T , Yamamoto S , Ugata Y , Sakakura K , Fujita H . Efficacy of an alternative positioning of intracardiac defibrillation catheters in atrial fibrillation ablation. J Arrhythmia. 2025;41(2):e70044. 10.1002/joa3.70044 PMC11926564 40123859

